# A Genetic Variant in the Promoter Region of miR-106b-25 Cluster and Risk of HBV Infection and Hepatocellular Carcinoma

**DOI:** 10.1371/journal.pone.0032230

**Published:** 2012-02-29

**Authors:** Yao Liu, Yixin Zhang, Juan Wen, Li Liu, Xiangjun Zhai, Jibin Liu, Shandong Pan, Jianguo Chen, Hongbing Shen, Zhibin Hu

**Affiliations:** 1 Department of Epidemiology and biostatistics, MOE Key Laboratory of Modern Toxicology, School of Public Health, Nanjing Medical University, Nanjing, China; 2 Department of Hepatobiliary Surgery, Nantong Tumor Hospital, Nantong, China; 3 Institute of Digestive Endoscopy and Medical Center for Digestive Diseases, the Second Affiliated Hospital of Nanjing Medical University, Nanjing, China; 4 Department of Infection Diseases, Jiangsu Province Center for Disease Prevention and Control, Nanjing, China; 5 Qidong Liver Cancer Research Institute, Qidong, China; 6 Section of Clinical Epidemiology, Jiangsu Key Lab of Cancer Biomarkers, Prevention and Treatment, Cancer Center, Nanjing Medical University, Nanjing, China; 7 State Key Laboratory of Reproductive Medicine, Nanjing Medical University, Nanjing, China; University of Montreal, Canada

## Abstract

**Background:**

MiR-106b-25 cluster, hosted in intron 13 of *MCM7*, may play integral roles in diverse processes including immune response and tumorigenesis. A single nucleotide polymorphism (SNP), rs999885, is located in the promoter region of *MCM7*.

**Methods:**

We performed a case-control study including 1300 HBV-positive hepatocellular carcinoma (HCC) cases, 1344 HBV persistent carriers and 1344 subjects with HBV natural clearance to test the association between rs999885 and the risk of HBV persistent infection and HCC. We also investigated the genotype-expression correlation between rs999885 and miR-106b-25 cluster in 25 pairs of HCC and adjacent non-tumor liver tissues.

**Results:**

Compared with the HBV natural clearance subjects carrying rs999885 AA genotype, those with AG/GG genotypes had a decreased risk of chronic HBV infection with an adjusted odds ratio (OR) of 0.79 [95% confidence intervals (CIs) = 0.67–0.93]. However, the AG/GG genotypes were significantly associated with an increased HCC risk in HBV persistent carriers (adjusted OR = 1.25, 95% CIs = 1.06–1.47). Expression analysis revealed that the expression level of miR-106b-25 cluster was significantly higher in AG/GG carriers than those in AA carriers in non-tumor liver tissues.

**Conclusions:**

These findings indicate that the A to G base change of rs999885 may provide a protective effect against chronic HBV infection but an increased risk for HCC in HBV persistent carriers by altering the expression of the miR-106b-25 cluster.

## Introduction

Hepatocellular carcinoma (HCC) is one of the major cancer burdens in China [1]. Hepatitis B virus (HBV) infection has been well established as a risk factor for liver carcinogenesis [2]. HBV persistent infection or HBV natural clearance is influenced by complex factors of viral, host age, environmental and genetic makeup, while hereditary factors may also play critical roles in the pathogenesis of HCC, together with other environmental factors, such as cigarette smoking, alcohol drinking and aflatoxins ingestion [3], [4].

MicroRNAs (miRNAs) are small non-coding RNAs that may regulate genes expression, either by inhibiting target mRNA translation or inducing its degradation [5]. Alterations of miRNA, including expression disorders and mutations, are involved in the initiation and progression of human cancers [6]. Accumulating data revealed that a subset of miRNAs deregulated in HCC [7], [8], [9], [10], [11]. Among them, miR-106b-25 cluster, including miR-106b, miR-93 and miR-25, is of particular interest, for its integral roles in diverse processes including immune response and tumorigenesis [11], [12], [13]. The cluster is hosted in intron 13 of *MCM7*, the abbreviation of minichromosome maintenance complex component 7, and Petrocca *et al* indicated that its transcription might be driven by the host gene [14]. We searched single nucleotide polymorphisms (SNPs) in the promoter region of *MCM7* with the criteria of minor allele frequency (MAF)>0.05 in Han Chinese and found two SNPs(rs4319008 and rs999885) in high linkage disequilibrium (LD) (r-square = 1). We chose rs999885 and performed a case-control study including 1300 HBV positive HCC cases, 1344 HBV persistent carriers and 1344 HBV natural clearance subjects to test the association between this SNP and risk of HBV persistent infection and HCC.

## Methods

### Study Subjects

This case-control study was approved by the institutional review board of Nanjing Medical University. The subjects' enrollment was described previously [15]. In brief, the newly diagnosed HCC patients were consecutively recruited from January 2006 to December 2010 at the Nantong Tumor Hospital, the Qidong liver cancer institute of Jiangsu Province and the First Affiliated Hospital of Nanjing Medical University, Jiangsu, China. The controls were screened for the HBV/HCV markers from two cities in Jiangsu Province (9720 persons from Changzhou and 48422 persons from Zhangjiagang) in 2004 and 2009, respectively. About 865 (8.9%) HBV persistent carriers and 1759 (18.1%) subjects with HBV natural clearance were identified from Changzhou; while 2156 (4.5%) HBV persistent carriers and 7851 (16.2%) subjects with HBV natural clearance were identified from Zhangjiagang. We randomly selected 1344 HBV persistent carriers and 1344 HBV natural clearance people from the two cities and matched to the HCC cases on age and gender. HBV persistent carriers were positive for both HBV surface antigen (HBsAg) and antibody to hepatitis B core antigen (anti-HBc), negative for HCV antibody (anti-HCV). Subjects with HBV natural clearance were negative for HBsAg and anti-HCV, plus positive for both antibody to hepatitis B surface antigen (anti-HBs) and anti-HBc.

### Serological testing

HBsAg, anti-HBs, anti-HBc and anti-HCV were detected by the enzyme-linked immunosorbent assay (Kehua Bio-engineering Co., Ltd., Shanghai, China) in the serum following the manufacturer's instructions as described previously [15].

### SNPs Genotyping

Genomic DNA was extracted from a leukocyte pellet by traditional proteinase K digestion, phenol-chloroform extraction and ethanol precipitation. The SNP, rs999885 A>G was genotyped by using the TaqMan allelic discrimination assay on a 7900 system (Applied Biosystems). The primers and probes for rs999885 were designed as follows: Primers: sense, F5′-CATCCAAAGCAATCAATCATCAG, antisense, 5′-GGCTTGGTCAGTAGAGGGAAAG; Probes: allele G, FAM-CCCTCTTCTCTTTC-MGB, allele A, HEX-CCCTCTTTTCTTTC-MGB. The genotyping was performed without knowing the subjects' case or control status. Two blank (water) controls in each 384-well plate were used for quality control and more than 5% samples were randomly selected and repeated, yielding a 100% concordant.

### Tissue Samples

We collected 25 pairs of HCC and adjacent non-tumor liver tissues from the patients who had undergone surgery between April 2008 and October 2010 from the Nantong Tumor Hospital. All cases were histopathologically diagnosed as HCC and had no radiotherapy or chemotherapy before surgical operation.

### Quantitative Reverse Transcriptase–Polymerase Chain Reaction (RT-PCR)

**Figure 1:**
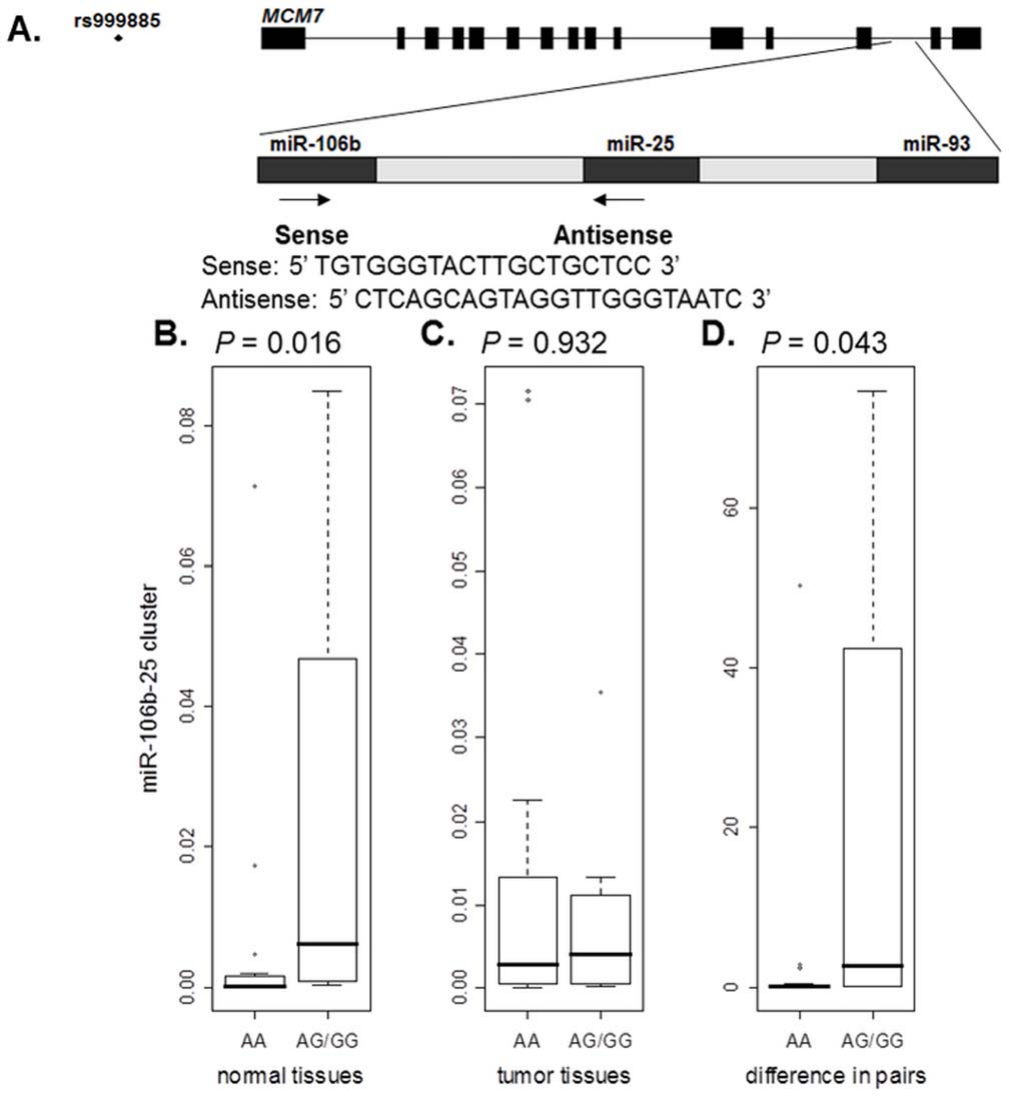
Genotype–expression correlations for rs999885 and miR-106b-25 cluster expression levels. (A) The positions and sequences of the primers used for RT-PCR to quantify the level of miR-106b-25 cluster are indicated. (B, C) Quantitative RT-PCR was used to measure expression levels of miR-106b-25 cluster in 25 pairs of HCC and adjacent non-tumor liver tissues. Normalization was performed by the β-actin. Genotype–expression correlations for genotypes and expression levels of miR-106b-25 cluster in normal and tumor tissues, respectively, which were calculated by using the equation 2^−ΔCt^, and ΔCt = Ct_normal_(Ct_tumor_)−Ct_β-actin-normal_(Ct_β-actin-tumor_). (D) Genotype–expression correlation for rs999885 and the expression level of miR-106b-25 cluster, which was calculated by using the equation 2^−ΔΔCt^, and ΔΔCt = (Ct_normal_−Ct_β-actin-normal_)−(Ct_tumor_−Ct_β-actin-tumor_).

Quantitative RT-PCR was performed to determine the expression level of miR-106b-25 cluster with Power SYBR Green PCR Master Mix (Applied Biosystems Inc.) and the primers were shown in Fig. 1A. Normalization was performed with β-actin (sense, 5′- AGAAAATCTGGCACCACACC -3′, antisense, 5′- GGGGTGTTGAAGGTCTCAAA -3′). All PCR reactions, including no-template controls and real-time minus controls, were performed in triplicate. A relative expression was calculated using the equation 2^−ΔCt^, in which ΔCt = Ct_gene_−Ct_β-actin_, and also the equation 2^−ΔΔCt^, in which ΔΔCt = (Ct_normal_−Ct_β-actin-normal_)−(Ct_tumor_−Ct_β-actin-tumor_).

### Statistical Analysis

The Student's t-test and χ^2^ test were used to detect differences of demographic characteristics, genotype frequencies of the SNP between the cases and controls for continuous variables and categorical variables, respectively. Associations between the genotypes and risk of HCC or HBV persistent infection were estimated by computing odds ratios (ORs) and their 95% confidence intervals (CIs) from logistic regression analyses. The crude ORs were calculated from univariate logistic regression, while the adjusted ORs were from multivariate logistic regression with the adjustment for age, gender, drinking and smoking status. The Chi-square-based *Q* test was applied to test the heterogeneity of associations between subgroups. Expression levels of miR-106b-25 between different groups were compared by the Mann-Whitney U test. All of the statistical analyses were performed with R software (version 2.13.0; The R Foundation for Statistical Computing). All tests were two-sided.

## Results

The demographic characteristics of the 1300 HBV positive HCC cases, 1344 HBV persistent carriers and 1344 persons with HBV natural clearance have been summarized previously [15]. Briefly, no significant differences were detected in the age and gender distributions between the three groups (*P* = 0.839 and 0.716, respectively). Smoking rates were also similar between the three groups. However, the drinking rates were higher among cases than that in controls (*P*<0.001 for both HCC cases compare to HBV persistent carriers and clearance controls).

The genotyping call rate for rs999885 was 98.65%. The genotype distributions of rs999885 in HBV positive HCC cases, HBV persistent carriers and HBV natural clearance subjects are shown in Table 1. In the logistic regression analyses between HBV persistent carriers and HBV natural clearance subjects, it was shown that the variant genotypes (AG/GG) of rs999885 were associated with a significantly decreased risk for chronic HBV infection (adjusted OR = 0.79, 95% CIs = 0.67–0.93, *P* = 0.004) in the dominant genetic model. However, compared with the HBV persistent carriers carrying wild-type AA of rs999885, those with the AG genotype had an increased risk for HCC with adjusted OR of 1.28 (95% CIs = 1.08–1.52), and the AG/GG genotypes had an increased risk by 25% (adjusted OR = 1.25, 95% CIs = 1.06–1.47, *P* = 0.008) (Table 1).

**Table 1:** Genotype frequencies of rs999885 and susceptibility of HCC and HBV persistent infection.

Genotype	HCC patients	HBV persistent carriers	HBV natural clearance subjects	OR(95%CI)^a^	*P*	OR(95%CI)^b^	*P*
	(N = 1300)	(N = 1344)	(N = 1344)				
rs999885	n %	n %	n %				
AA	815(63.1)	902(68.0)	823(62.6)	1		1	
AG	439(34.0)	381(28.7)	454(34.5)	1.28(1.08–1.52)		0.77(0.65–0.90)	
GG	38(2.9)	44(3.3)	38(2.9)	0.96(0.61–1.51)		1.06(0.68–1.66)	
AG/GG	477(36.9)	425(32.0)	492(37.4)	**1.25(1.06–1.47)**	**0.008**	**0.79(0.67–0.93)**	**0.004**

NOTE: Multivariate logistic regression analyses adjusted for age, sex, smoking status and drinking status.

a HCC patients *vs.* HBV persistent carriers.

b HBV persistent carriers *vs.* HBV natural clearance subjects.

The association between rs999885 and the susceptibility to HCC and HBV persistent infection was also evaluated by stratifying on age, gender, drinking and smoking status (Table S1). However, no significant heterogeneity was detected between the subgroups, implying independent genetic effect of rs999885.

To characterize the functional relevance of the SNP rs999885, we conducted a correlation analysis between the rs999885 genotypes and the expression levels of miR-106b-25 cluster. We found that, in the 25 pairs of tissue specimens, 8 had the rs999885 AA genotype, 16 with the AG genotype, and 1 with the GG genotype. As shown in Fig. 1B, the expression levels of miR-106b-25 were significantly higher in AG/GG carriers than those in AA carriers in normal liver tissues (0.024±0.033 for AG/GG *vs.* 0.006±0.017 for AA, *P* = 0.016). Furthermore, as shown in Fig. 1D, the relative expression levels between HCC and normal liver tissues of miR-106b-25 were also higher in AG/GG carriers than those in AA carriers (20.633±30.152 for AG/GG *vs.* 3.394±12.090 for AA, *P* = 0.043).

## Discussion

In this study, we investigated the association between rs999885 and the susceptibility to HBV persistent infection and HCC in a Chinese population. We found that the A to G base change of rs999885 demonstrated protective effect on chronic HBV infection, but increased the risk of HCC in HBV persistent carriers.

To date, accumulating data has showed that miR-106b-25 cluster plays oncogenic roles in cancers, through influencing tumor growth, cell survival, and angiogenesis [16], [17], [18]. This cluster has been reported to be up-regulated in several cancers, including esophageal adenocarcinoma [18], gastric cancer [19], prostate cancer [20], head and neck squamous cell carcinoma [21], and HCC [11]. Specially, by sequencing analysis, it was shown that the expression levels of miR-106b, miR-93 and miR-25 were higher in liver cancer cell lines than those in normal liver cells [22]. In this study, we found that the expression levels of miR-106b-25 were significantly higher in AG/GG carriers, which was consistent with the risk effect of the variant genotypes. However, reverse findings were reported in immune disorders. De Santis *et al* found that miR-106b-25 down-regulated in multiple sclerosis patients compared to healthy donors in specific T regulatory cells [23], which might support the protective role of the variant genotypes of rs999885 on susceptibility to chronic HBV infection. In addition, several independent studies also revealed that miR-106b-25 cluster promoted cell cycle progression by suppressing the Cip/Kip family members of Cdk inhibitors, in which miR-25 targeted p57, while miR-106b and miR-93 controlled p21 [17], [24], [25]. Moreover, the miR-106b-25 cluster may resist the TGF-β tumor suppression pathway [26], and miR-93 alone may promote tumor growth and angiogenesis by suppressing integrin-β8 expression [16].

The miR-106b-25 polycistron is activated by genomic amplification and over-expression of MCM7 [27], and the miR-106b-25 cluster may cooperate with its host gene *MCM7* in cellular transformation [28]. The SNP rs999885 (A>G) is located at 1749-bp upstream from transcriptional start position of *MCM7*. According to the web-based SNP analysis tool (TFSEARCH 1.3), the A allele of rs999885 may have high affinity with a transcription factor, sex-determining region Y protein (SRY), which involved in gene regulation including promoter activation or repression depending on its interacted protein [29], [30]. In the current study, we provided evidence that the rs999885 variant could influence miR-106b-25 expression.

In summary, this study, with a relative large population, showed that a genetic variant in the promoter region of miR-106b-25 cluster might provide a protective effect against chronic HBV infection but an increased risk for HCC in HBV persistent carriers by affecting the expression of miR-106b-25 cluster. Further studies incorporating diverse populations and functional assays are warranted to validate and extend our findings.

## Supporting Information

Table S1 **Stratified analyses on association between rs999885 and risk of HCC and HBV persistent infection.** NOTE: Multivariate logistic regression analyses adjusted for age, sex, smoking status and drinking status in dominant genetic model (excluded the stratified factor in each stratum). ^a^HCC patients *vs.* HBV persistent carriers. ^b^HBV persistent carriers *vs.* HBV natural clearance subjects. ^c^ *P* for heterogeneity.
(DOC)
