# Supplementary material for: A Genetic Variant in the Promoter Region of miR-106b-25 Cluster and Risk of HBV Infection and Hepatocellular Carcinoma
Source: PLoS One. 2012 Feb 29;7(2):e32230. doi: 10.1371/journal.pone.0032230 (PMC3290543; doi:10.1371/journal.pone.0032230)
Supplement: Table S1 — Stratified analyses on association between rs999885 and risk of HCC and HBV persistent infection. NOTE: Multivariate logistic regression analyses adjusted for age, sex, smoking status and drinking status in dominant genetic model (excluded the stratified factor in each stratum). aHCC patients vs. HBV persistent carriers. bHBV persistent carriers vs. HBV natural clearance subjects. c P for heterogeneity. (DOC) [file pone.0032230.s001.doc]

**Table S1** Stratified analyses on association between rs999885 and risk of HCC and HBV persistent infection

| Variables | HCC/ Carriers / Clearances | | OR(95%CI) ^a^ | *P* ^c^ | OR(95%CI) ^b^ | *P* ^c^ |
| --- | --- | --- | --- | --- | --- | --- |
|  | AA | AG+GG |  |  |  |  |
| Age |  |  |  |  |  |  |
| ≤53 | 437(63.3)/469(67.9)/433(62.9) | 253(36.7)/222(32.1)/255(37.1) | **1.27(1.01-1.59)** | 0.887 | 0.82(0.65-1.02) | 0.706 |
| >53 | 378(62.8)/433(68.1)/390(62.2) | 224(37.2)/203(31.9)/237(37.8) | 1.24(0.98-1.58) |  | **0.77(0.61-0.98)** |  |
| Gender |  |  |  |  |  |  |
| Male | 699(63.6)/755(67.1)/695(63.1) | 400(36.4)/370(32.9)/407(36.9) | 1.18(0.99-1.41) | 0.133 | 0.84(0.70-1.00) | 0.112 |
| Female | 116(60.1)/147(72.8)/128(60.1) | 77(39.9)/55(27.2)/85(39.9) | **1.73(1.09-2.77)** |  | **0.58(0.38-0.88)** |  |
| Smoking status |  |  |  |  |  |  |
| Ever | 489(63.4)/513(67.5)/476(62.2) | 282(36.6)/247(32.5)/289(37.8) | 1.22(0.98-1.52) | 0.711 | **0.79(0.64-0.98)** | 0.939 |
| Never | 326(62.6)/389(68.6)/347(63.1) | 195(37.4)/178(31.4)/203(36.9) | **1.30(1.01-1.68)** |  | 0.78(0.61-1.00) |  |
| Drinking status |  |  |  |  |  |  |
| Ever | 481(63.2)/409(68.3)/346(61.9) | 280(36.8)/190(31.7)/213(38.1) | 1.24(0.99-1.57) | 0.925 | **0.76(0.60-0.97)** | 0.646 |
| Never | 334(62.9)/493(67.7)/477(63.1) | 197(37.1)/235(32.3)/279(36.9) | 1.26(0.99-1.61) |  | 0.82(0.66-1.02) |  |
